# The Small-Pox Outbreak

**Published:** 1895-03

**Authors:** 


THE SMALL-POX OUTBREAK.—Small-pox made its appearance in several places in Texas recently, almost simultaneously. It was reported at Wortham, in Freestone county, Richland, in Navarro county, Fort Worth, and other points, a few days after it was first reported at Taylor. The manner in which it originated emphasizes the necessity not only for a general and extensive vaccination of all non-immune persons and revaccination of many others, and also for a law, rigidly enforced, in every town and city, requiring every householder, and every physician especially, and in fact all persons who have knowledge of the presence of any case of contagious disease, to report it at once to the proper authorities, under heavy penalty for failure.

The circumstances were these: A man named Straub kept an eating house of some kind at Taylor. He fed a Mexican, and in default of payment, *took his blanket!* Straub's child—a boy—luxuriated in this tropical delicacy, and caught small-pox. (This would look like "poetic justice" if it were not for the fact that the boy, and not the avaricious father, suffered, and the Mexican also,--the innocent cause of it: he froze to death, we suppose, as it was during the ten days' blizzard.) The boy continued to go to school till the disease broke out in him, and then his father put him to bed in a shed-room adjoining the apartment where he fed citizens and transients, daily, and concealed the nature of the malady. Meantime, there is no telling how many persons were exposed, nor the extent to which the infection has been scattered. In a short time afterwards the disease is reported at the points above mentioned, and elsewhere, and "quarantine" is the order of the day. State Health Officer Swearingen visited all of the infected points, and the local medical officers have the disease under control. As soon as a case is developed, it is at once isolated and put under guard, and all who are known to have been exposed are also separated and kept under observation till the danger is passed; are disinfected, clothing changed, etc.; and after death, or recovery of a case, all the necessary sanitary measures are invoked to prevent spread. Meantime, general vaccination is urged, and is practiced by the more intelligent part of each community. But for the concealment, and the fact that many who were exposed got away, there would be no danger of a spread. As it is, wherever one develops the disease, if isolation both of patient and exposed, be promptly done, there need not occur any other cases. There is no necessity for one town to quarantine against another, unless the disease should become epidemic,—of which there is no danger if the sanitary officers do their duty; the quarantining should be confined to the disease; shut it up, allow no communication, then there is no necessity of shutting it *out*.

This brings up the subject of compulsory vaccination. It has long been a disputed question whether any government, State or municipal, has the right to compel vaccinations. It is claimed by those opposed to vaccination, that it is an invasion of personal rights, and is unconstitutional. We do not see, in this enlightened age, and in the light of the very evident fact, that vaccination will prevent small-pox, and that nothing else will, how any intelligent person can object to it. They take fearful risks, and are, sooner or later caught, if they travel. It would appear to us that the State, undoubtedly, has the right to compel vaccination as a police measure for the public safety. In the days during the war and just after, when there was so much spurious vaccine matter, and it was run through several systems—often producing results nearly as bad as small-pox itself, one could, with a show of reason, object to having one's family vaccinated; but now all matter sold is guaranteed, and at the worst it can produce only a little excitement, perhaps fever and headache—and whether constitutional or not, every non-protected person should be compelled to be vaccinated. It is suggested, as a means of obviating the "constitutional" objection, that evidence of recent successful vaccination be made a *sine qua non* to any child entering one of the public schools. Teachers of private schools also could make it a condition. In fact, the legislature should pass an act requiring something of this kind attached to the charter of every town or city. When vaccination becomes general, and not till then, will small-pox disappear from terra firma.

We had *nearly* said "stamped out"; that is a figure of speech we much object to; it is played out; it is obsolete; it has lost its meaning and application,—if it ever had any,—yet everybody seems to understand what is meant by it. No one "stamps" upon a case of small-pox; no one "stamps" upon anything that I know of. It is assumed that there is a resemblance to a fire, and that in the beginning a fire may be extinguished by stamping upon it, but even this—the only shadow of a simile that I can see, is pretty far fetched. Let us say, of small-pox, suppressed, exterminated. It is like another figure of speech, much abused; that is, of victory "perching upon a banner," success "crowning one's efforts." How can victory "perch" on anything? Let us have a new form for expressing these ideas.

